# Thermal- and Light-Induced Evolution of the 2D/3D Interface in Lead-Halide Perovskite Films

**DOI:** 10.1021/acsami.1c09695

**Published:** 2021-09-29

**Authors:** Francesca Fiorentino, Munirah D. Albaqami, Isabella Poli, Annamaria Petrozza

**Affiliations:** † Center for Nano Science and Technology @PoliMi, Istituto Italiano di Tecnologia, via G. Pascoli 70/3, 20133 Milano, Italy; ‡ Physics Department, Politecnico di Milano, Piazza L. da Vinci, 32, 20133 Milano, Italy; § Chemistry Department, College of Science, King Saud University, Riyadh 11451, Saudi Arabia

**Keywords:** 2D/3D perovskites, phenethylammonium ion, photoluminescence, thermal stress, moisture instability, photostability, halide perovskite degradation

## Abstract

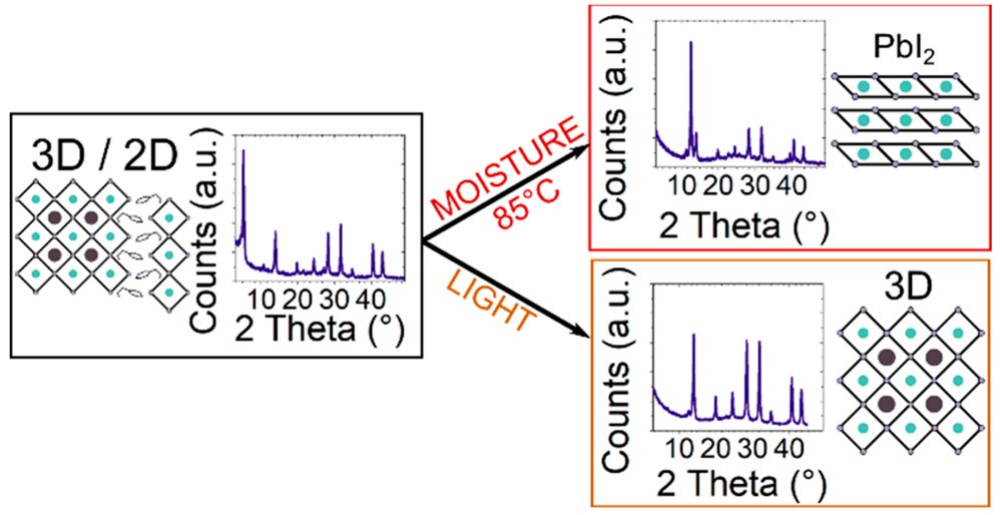

The instability of halide perovskites toward moisture is one of the main challenges in the field that needs to be overcome to successfully integrate these materials in commercially viable technologies. One of the most popular ways to ensure device stability is to form 2D/3D interfaces by using bulky organic molecules on top of the 3D perovskite thin film. Despite its promise, it is unclear whether this approach is able to avoid 3D bulk degradation under accelerated aging conditions, i.e., thermal stress and light soaking. In this regard, it is crucial to know whether the interface is structurally and electronically stable or not. In this work, we use the bulky phenethylammonium cation (PEA^+^) to form 2D layers on top of 3D single- and triple-cation halide perovskite films. The dynamical change of the 2D/3D interface is monitored under thermal stress and light soaking by in situ photoluminescence. We find that under pristine conditions the large organic cation diffuses only in 3D perovskite thin films of poor structural stability, i.e., single-cation MAPbI_3_. The same diffusion and a dynamical change of the crystalline structure of the 2D/3D interface are observed even on high-quality 3D films, i.e., triple-cation MAFACsPbI_3_, upon thermal stress at 85 °C and light soaking. Importantly, under such conditions, the resistance of the thin film to moisture is lost.

## Introduction

Perovskite solar cells (PSCs) are among the most promising emergent photovoltaic technologies. Solution-processed devices have now reached power conversion efficiencies (PCEs) higher than 25^1^ and 18%^2,3^ on small cells and mini-modules, respectively. Despite the great promise and strengths of PSCs, some concerns regarding their long-term stability under real-world conditions still hold the technology back from its commercialization.^4^ Therefore, most recent works have focused on understanding degradation mechanisms occurring in PSCs to improve their stability.

Halide perovskites in their 3D form have a ABX_3_ crystal structure, where A is an organic/inorganic cation (for example, methylammonium MA^+^, formamidinium FA^+^, or Cs^+^), B is a metal cation (Pb^2+^ or Sn^2+^), and X is a halide (Cl^–^, Br^–^, or I^–^).^5^ Alternative to 3D perovskites, 2D perovskites with the so-called Ruddlesden–Popper structure R_2_A_*n*–1_B_*n*_X_3*n*+1_ have been studied, where R is a bulky organic cation that acts as a spacer between the inorganic sheets and *n* indicates the number of inorganic layers held together.^6^ They show wider bandgaps and higher exciton binding energies, which reduce the light absorption spectra and hinder the separation of carriers, respectively. As a consequence, they generally present lower PCEs than 3D perovskite when embodied in solar cells.^7^ Nevertheless, one of the most studied approaches to improve the stability of PSCs consists of combining 3D perovskites with 2D structures, by forming either a layered structure, where 3D frameworks are sliced into well-defined 2D layers,^8−10^ or a multijunction stack, where the 2D perovskite is formed only on the top surface of a 3D perovskite film.^11,12^ One-step and layer-by-layer growth techniques have been shown, where the organic bulky spacer is either directly blended within the perovskite precursor solution or deposited over the bulk 3D perovskite layer, respectively. Both approaches showed that the low-dimensional perovskite layer self-assembles as a thin capping layer on the top of the bulk 3D perovskite.^13^ It was demonstrated that such thin film not only hinders moisture uptake but can also act as a selective charge extraction layer, reducing the recombination of photogenerated carriers at the interface, enhancing charge-transfer kinetics, and inhibiting the volatilization of methylammonium.^14−20^ To date, the highest efficiency reported for 2D/3D junction absorbers is 23.32%, where a phenetylammonium (PEA) salt solution is spin-coated onto a FAMAPbI_3_ perovskite film.^12^

Given the great advances in engineering multijunction structural perovskite absorbers, a complete understanding of the 3D/2D interface evolution under external stressors like heat, illumination, and continuous biasing is needed for considering this solution in real life applications. For example, it is not clear whether the 2D perovskite layer is stable on the surface without undergoing a structural transformation. Indeed, most of the stability tests reported in the literature for 3D/2D multijunction PSCs have been measured either on encapsulated devices or in an inert atmosphere at room temperature, while internationally recognized qualification standards require the solar cell to be aged at 85 °C under continuous maximum power point tracking at 1 sun illumination.^21^ Liu et al. recently showed that the PCE of MAPbI_3_ solar cells capped with 2D BA_2_Pb_2_I_6_ thin films dropped by 40% after only 125 h of thermal aging at 85 °C in nitrogen, where BA stands for butylammonium.^22^ On the other hand, Sutanto et al. showed that the 2D structure of PEA_2_PbI_4_ deposited on top of a triple-cation 3D perovskite film undergoes a dynamical transformation upon thermal aging at 50 °C that causes a decrease in PCE of 2D/3D perovskite solar cells after only 100 min of heating, reaching 97% of the initial PCE.^23^

In this work, we study 3D perovskite films with a 2D layer of PEA_2_PbI_4_ on the top surface and its evolution upon exposure to moisture, heat, and continuous illumination. We reveal that a 2D layer of PEA_2_PbI_4_ is formed uniquely on the top surface of triple-cation (FA_0.83_MA_0.17_)_0.95_Cs_0.05_Pb(I_0.83_Br_0.17_)_3_ 3D perovskite films. In contrast, when MAPbI_3_ perovskite films are used—showing a lower PL quantum yield which we associate to a higher defectivity of the thin film—not all PEAI self-assembles as PEA_2_PbI_4_ on the surface. Part of the added PEAI diffuses through the bulk forming PEA_2_PbI_4_ phases within the bulk even before thermal aging. Such diffusion is observed on triple-cation (FA_0.83_MA_0.17_)_0.95_Cs_0.05_Pb(I_0.83_Br_0.17_)_3_ 3D perovskite films too when subjected to long-term thermal stress and light soaking. We further show that the 2D perovskite capping layer quickly disappears when the film is subjected to mild moisture and heat at the same time, allowing water molecules to penetrate through the film and convert 3D halide perovskite into PbI_2_.

## Experimental Section

### Materials and Methods

Lead(II) iodide (PbI_2_, 99.99%, CAS No. 10101-63-0) and lead(II) bromide (PbBr_2_, ≥98%) were purchased from Tokyo Chemical Industry (TCI); formamidinium iodide (FAI), phenethylammonium iodide (PEAI) methylammonium bromine (MABr), and methylammonium iodide (MAI) were purchased from Dyesol; and cesium iodide (CsI) was purchased from Alfa Aesar. *N*,*N*-Dimethylformamide (DMF, anhydrous, 99,8%), chlorobenzene (anhydrous, 99.8%), dimethyl sulfoxide (DMSO, anhydrous, ≥99.9%), and isopropyl alcohol (IPA) were purchased from Sigma-Aldrich. All chemicals were used without any further purification. Glass substrates were cleaned in an ultra bath sonicator with deionized (DI) water plus 3% volume of Hellmanex III, DI water, acetone, and IPA, for 10 min each step. The so-cleaned glass substrates were treated with oxygen plasma for 10 min just before any further deposition. All samples were prepared inside the glovebox under a controlled N_2_ atmosphere.

Pristine MAPbI_3_ thin film perovskites were prepared by spin-coating a 1.2 M solution (MAI:PbI_2_ = 1:1) in DMSO:DMF (volume ratio 1:4) at 4000 rpm for 30 s. After 10 s, 150 μL of chlorobenzene was dropped on the spinning sample. The precursor solution was left stirring overnight at room temperature prior to the deposition. An hour before deposition, the temperature was raised to 50 °C while the solution was left stirring. The sample was annealed for 10 min at 100 °C.

Pristine MAFACsPbI_3_ thin film perovskites were prepared by spin-coating a 1.3 M solution (FAI:MABr:PbI_2_ = 0.79:0.16:1) in DMSO:DMF (volume ratio 1:4) at 4000 rpm for 30 s. Six seconds before the end of the program, 200 μL of chlorobenzene was dropped on the spinning sample. The precursor solution was left stirring overnight at room temperature prior to the deposition. An hour before deposition, 5 mol % CsI solution (1.5 M in DMSO) was added and the temperature was raised to 50 °C while the solution was left stirring. The sample was annealed for 1 h at 100 °C.

2D/3D multijunction was created by dynamically spin-coating a PEAI solution (60 mmol in anhydrous IPA) on top of the precrystallized perovskite thin film at 4000 rpm for 30 s. The material was then post-annealed at 100 °C for 5 min to allow evaporation of the solvent. A 5% molar excess of PbI_2_ in the MAPbI_3_ (MAI:PbI_2_ = 1:1.05) and CsFAMAPb(IBr)_3_ (FAI:PbI_2_ = 1:1.05) precursor solution was added to obtain more reproducible samples with higher structural order of the 2D phases (Figure S1).

### Characterization

PL spectra under thermal aging were measured using the electroluminesce module of the Arkeo all-in-one measurement platform (CicciResearch). The emission was measured using a fiber coupled CCD spectrometer (300–1100 nm) upon single-wavelength excitation (405 and 520 nm depending on the material) in N_2_ or atmospheric air. Samples were held at 85 °C using a Peltier-based TEC module also under PL acquisition. Light soaking was provided by a white Illuminator LED module calibrated at 1 sun intensity. UV/vis absorption was measured on perovskite thin films deposited on bare glass using a UV/vis/NIR spectrophotometer Lambda 1050, PerkinElmer, in the wavelength range 400–900 nm and with a step size of 4 nm. XRD patterns were recorded with a Bruker D8 Advance diffractometer with Bragg–Brentano geometry equipped with a Cu Kα1 (λ = 1.5418 Å) anode, operating at 40 kV and 40 mA. All of the diffraction patterns were collected at room temperature, with a step size of 0.05 in symmetric scan reflection mode and an acquisition time of 1 s. Perovskite films were prepared on bare glass substrates.

## Results and Discussion

To get deeper insights into the stability of 2D perovskite capping layers on 3D bulks, we explore two different 3D compositions: single-cation MAPbI_3_ and triple-cation (FA_0.83_MA_0.17_)_0.95_Cs_0.05_Pb(I_0.83_Br_0.17_)_3_ (hereafter defined as MAFACsPbI_3_).

**Figure 1:**
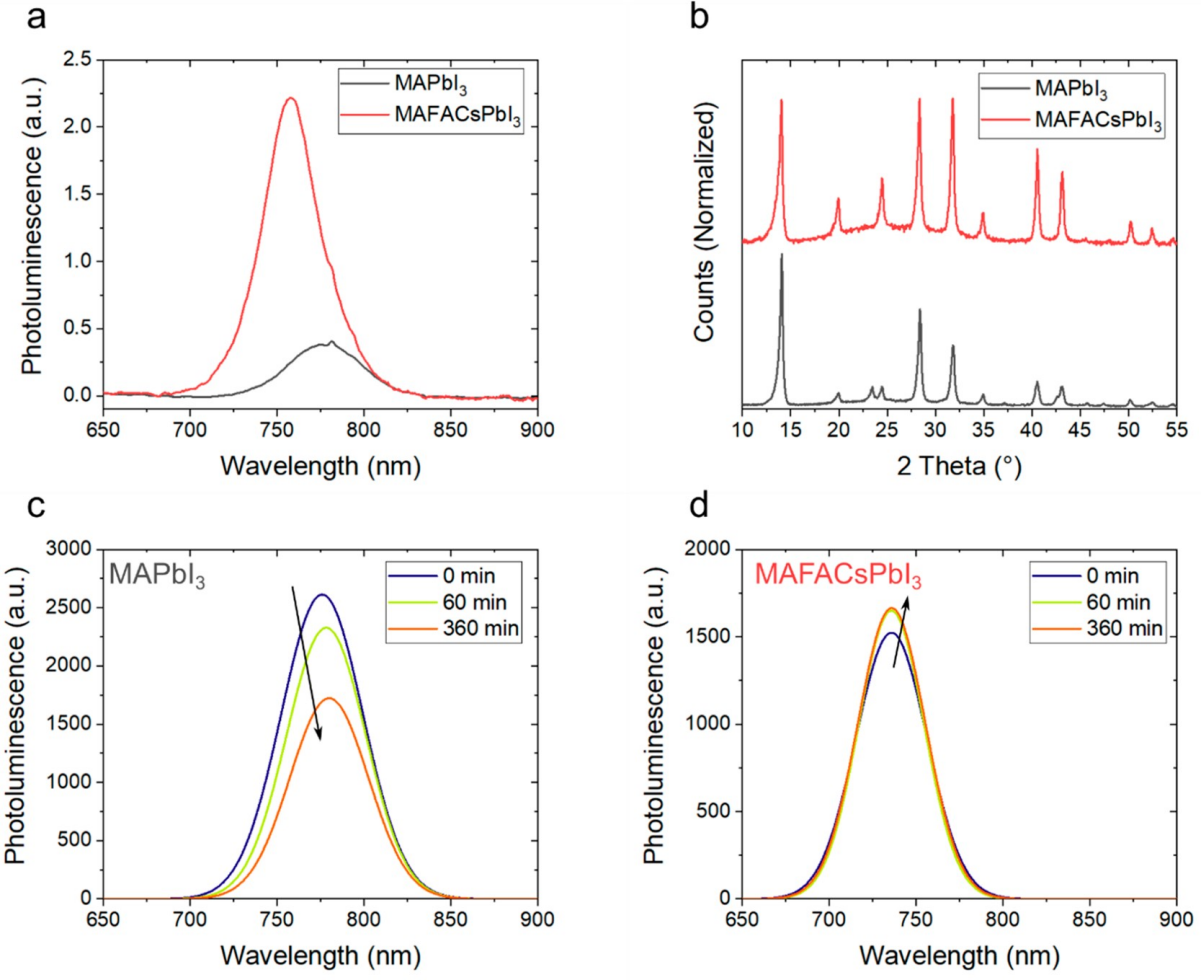
(a) PL spectra of pristine MAPbI_3_ and MAFACsPbI_3_ thin films measured under single-wavelength excitation of 520 nm in air (RH 65%). Spectra have been normalized with respect to the optical density of each film at the excitation wavelength. (b) XRD peaks of MAPbI_3_ and MAFACsPbI_3_. (c, d) Dynamical evolution of the PL spectrum of MAPbI_3_ and MAFACsPbI_3_ under thermal aging at 85 °C for 6 h in air (RH 65%), respectively (CW 520 nm excitation).

Figure 1a shows the photoluminescence (PL) spectra of pristine MAPbI_3_ and MAFACsPbI_3_, respectively (not subjected to any passivation process). The PL intensity is normalized with respect to the optical density measured at the excitation wavelength (520 nm) to ensure equal absorption of the films (UV–visible absorbance is shown in Figure S2). The photoluminescence of thin films is highly sensitive to defect density, which affects radiative and non-radiative carrier decay paths.^24^ The MAFACsPbI_3_ perovskite film is about 3 times more emissive than MAPbI_3_, inferring reduced non-radiative recombination. These results are in good agreement with previous works that attributed the role of cation mixing to the suppression of carrier recombination pathways and increased thermodynamic stability.^25−27^Figure 1b shows the X-ray diffraction (XRD) patterns for pristine MAPbI_3_ and MAFACsPbI_3_ thin films. Both materials exhibit good crystallinity, with main diffraction peaks at 14, 28, and 32° which correspond to the (110), (220), and (310) phases, respectively, typical of the tetragonal perovskite phase.

To further assess the stability of pristine MAPbI_3_ and MAFACsPbI_3_ under thermal stress, we monitored the PL spectra evolution while the samples were heated at 85 °C for 6 h in air, as shown in Figure 1c,d. The absolute intensity of the MAPbI_3_ 3D peak gradually decreases over 6 h of continuous exposure at 85 °C. In contrast, the intensity of the 3D peak of MAFACsPbI_3_ maintained its initial value, confirming the enhanced thermal stability of mixed cation structures and reduced formation of trap states.^28,29^ Notably, when the PL spectra were monitored in air at room temperature, both MAPbI_3_ and MAFACsPbI_3_ showed PL enhancement (Figure S3), probably due to passivation of trap states by oxygen.^30^ The photoluminescence results under thermal stress were confirmed also when MAPbI_3_ and MAFACsPbI_3_ thin films were aged at 85 °C in N_2_ for 250 h (Figure S4 and Figure S5). XRD patterns of MAPbI_3_ and MAFACsPbI_3_ were measured before and after thermal aging (Figure S6 and Figure S7); the intensity of XRD peaks associated with the 3D perovskite phase is reduced by 50 and 35% with respect to the initial values, respectively, indicating a more severe loss in crystallinity for MAPbI_3_ after thermal aging.

**Figure 2:**
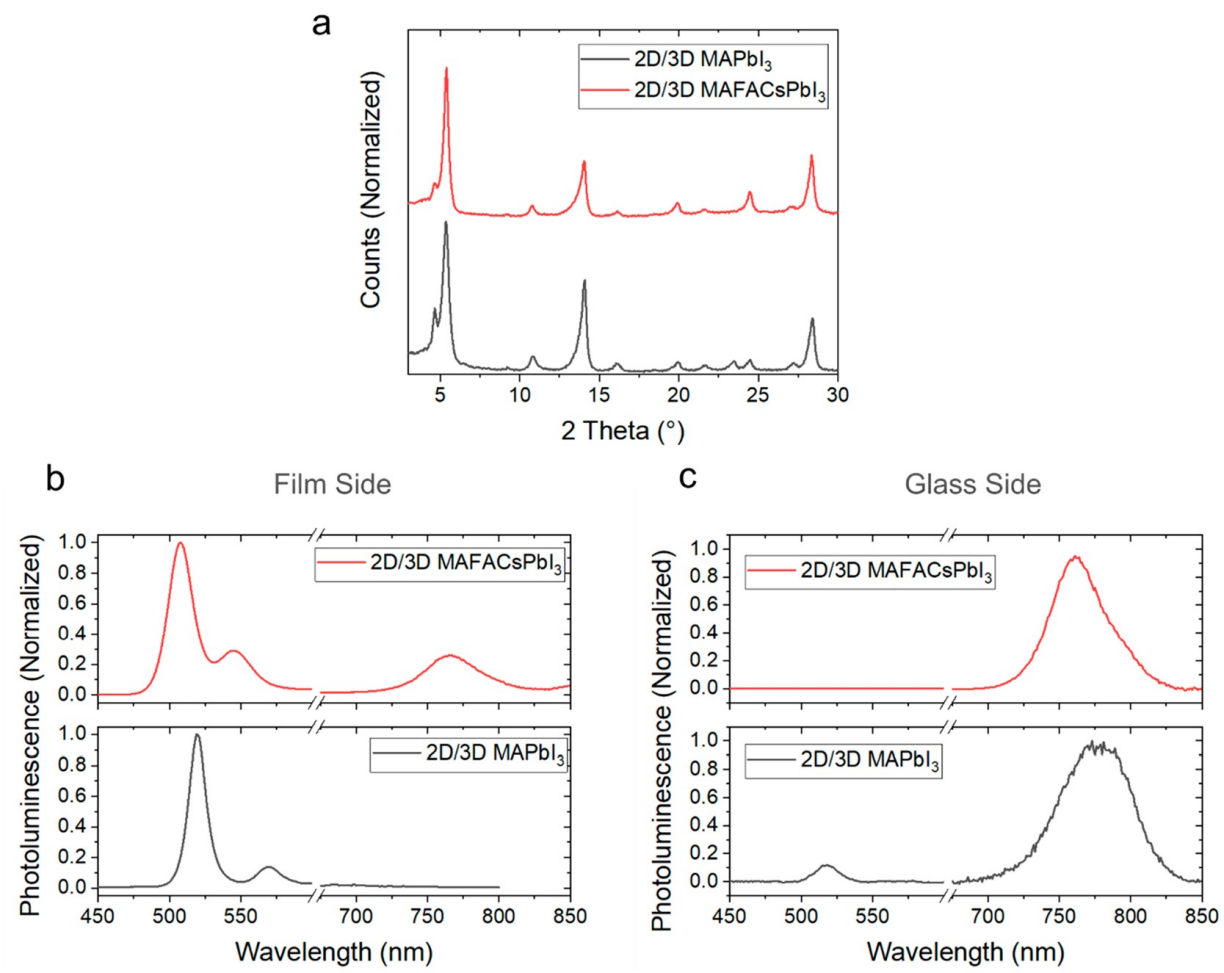
(a) XRD peaks of PEAI-treated MAPbI_3_ and PEAI-treated MAFACsPbI_3_ thin films after 10 min post-annealing at 100 °C. (b) PL spectra of PEAI-treated MAFACsPbI_3_ and PEAI-treated MAPbI_3_ measured by exciting the material from the film side. (c) PL spectra of PEAI-treated MAFACsPbI_3_ and PEAI-treated MAPbI_3_ measured by exciting the material from the glass side.

To create the multijunction 2D/3D thin films, PEAI was dissolved in isopropyl alcohol (IPA) and dynamically spin-coated on top of the 3D perovskite, which was deposited with 5 mol % excess of PbI_2_ to stabilize the interface.^18,31,32^Figure 2a shows the XRD patterns of PEAI-treated MAPbI_3_ and MAFACsPbI_3_ thin films after thermal annealing at 100 °C for 10 min. XRD peaks at 14, 28, and 5.4° can be clearly distinguished, which correspond to the tetragonal perovskite phase of the 3D bulk and, at lower angles, to the presence of PEAI. More specifically, the peak at 5.4° corresponds to the formation of pure 2D PEA_2_PbI_4_ perovskite. Weak XRD peaks at ∼4.7° can be distinguished in both materials, which are consistent with the diffraction peak of crystalline PEAI.^12^ The formation of crystalline PEAI has been previously reported in the literature, and it was found to gradually convert to a pure 2D perovskite phase during thermal annealing;^12,32^ therefore, we expect the presence of PEAI to reduce as the heating time increases.

Parts b and c of Figure 2 show the PL spectra of PEAI-treated MAFACsPbI_3_ and MAPbI_3_ measured by exciting the material from the film and glass side, respectively. When the PL spectra were recorded by exciting the materials from the film side, the emission from the 2D capping layer was mainly detected. Both spectra show a clear emission at around 520 nm and a weaker peak at around 560 nm, and MAFACsPbI_3_ only shows a clear PL peak from the 3D bulk perovskite at 760 nm. According to the literature, the bands appearing in the low-wavelength region correspond to the emission of PEA_2_MA_*n*–1_Pb_*n*_I_3*n*+1_, with *n* = 1 and *n* = 2, respectively.^23,33^ These results are in agreement with the XRD data shown in Figure 2a, where a preferential growth orientation of the pure 2D PEA_2_PbI_4_ phase is observed (5.4°). PL peaks from PEA_2_MA_*n*–1_Pb_*n*_I_3*n*+1_ on to MAFACsPbI_3_ are blue-shifted with respect to the ones observed in PEAI-treated MAPbI_3_, probably due to some I^–^ ions that have been replaced by Br^–^ ions.^34,35^Figure 2c shows that, when the PL is measured by exciting the PEAI-treated MAFACsPbI_3_ film through the glass, only the peak centered at 760 nm is clearly distinguished and no PL emissions in the low-wavelength region are observed, inferring that the 2D layer of PEA_2_PbI_4_ firmly stays on the top surface, in agreement with many reports in the literature.^13,32^ In contrast, when the PL spectrum of the PEAI-treated MAPbI_3_ film was measured from the glass side, as shown in Figure 2c, clear emission from the PEA_2_PbI_4_ was detected at 520 nm, suggesting that some of the PEAI might have diffused through the 3D bulk. These results show that, depending on the structural stability, and probably the defectivity of the underneath 3D bulk, PEAI may diffuse through the material, forming quasi-2D structures close to the substrate’s interface. The thermodynamic stability of the perovskite film is enhanced in mixed-cation compositions rather than single-cation ones due to easier deprotonation of MA.^26,36^ PEA^+^ may interact more easily with the PbI_6_ octahedra in MAPbI_3_ rather than MAFACsPbI_3_, due to weaker bonds between MA^+^ and the PbI_6_, causing less adhesion of PEA_2_PbI_4_ onto the surface and more facile diffusion of PEAI through the film during thermal annealing at 100 °C. Therefore, the robustness of the 2D overlayer may depend not only on the bulky molecule used to create the 2D/3D interface as previously reported^18^ but also on the quality of the underlying 3D bulk perovskite.

**Figure 3:**
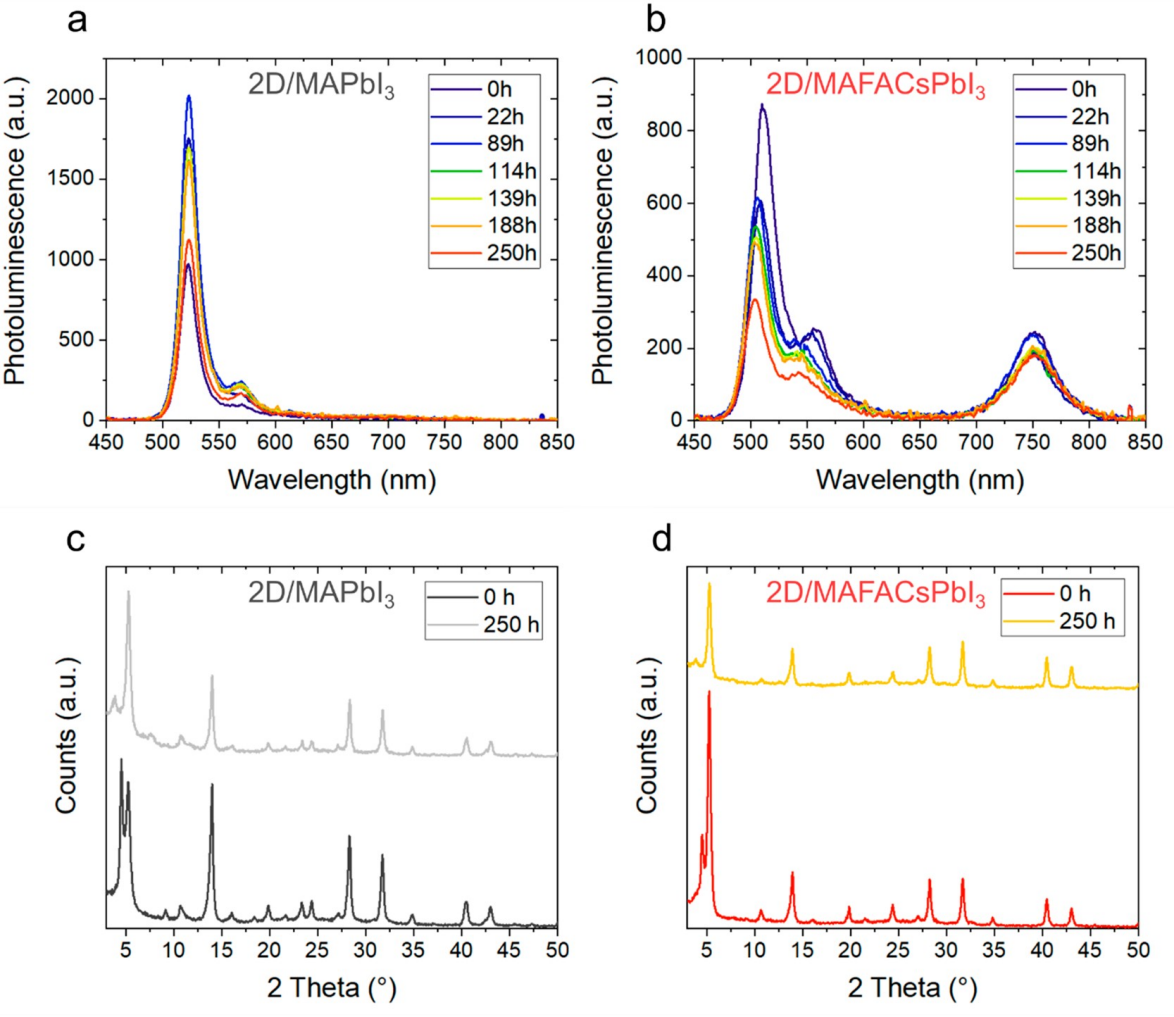
(a, b) PL spectra evolution of PEAI-treated MAPbI_3_ and PEAI-treated MAFACsPbI_3_ thin films upon thermal aging at 85 °C for 250 h in N_2_ (single-wavelength excitation at 405 nm), respectively. (c, d) XRD peaks of PEAI-treated MAPbI_3_ and PEAI-treated MAFACsPbI_3_ thin films before and after thermal aging at 85 °C in N_2_ for 250 h, respectively.

Then, we have investigated the evolution of the 2D/3D junction under thermal stress. Parts a and b of Figure 3 show the evolution of PL spectra of PEAI-treated MAPbI_3_ and PEAI-treated MAFACsPbI_3_ thin films under thermal stress (85 °C for 250 h, in N_2_). Upon thermal exposure, the 2D and quasi-2D PEA_2_MA_*n*–1_Pb_*n*_I_3*n*+1_ phases of both MAPbI_3_ and MAFACsPbI_3_ samples do not show an appreciable change in the emission peak position. The absolute intensity of the PEA_2_PbI_4_ (*n* = 1) PL peak formed onto MAFACsPbI_3_ diminishes over time, while the PL peak assigned to the 3D bulk emission at 760 nm remains unaltered upon thermal exposure, as also observed in pristine MAFACsPbI_3_ (Figure S5). Sutanto et al. observed similar trends for PEAI 2D/3D triple-cation perovskites upon thermal aging at 50 °C for 6 h. They suggest a dynamical structural variation of the 2D layer upon thermal stress, which does not affect the underneath 3D perovskite.^23^ In contrast, the absolute intensity of the PEA_2_PbI_4_ (*n* = 1) PL peak formed onto MAPbI_3_ shows an initial increase, followed by a subsequent decay. The segregation of PbI_2_ as a separate phase, a consequence of thermal decomposition,^37^ could explain the initial increase of the 2D PEA_2_PbI_4_ phase observed in PEAI-treated MAPbI_3_ films due to a reaction of the unconverted crystalline PEAI and unreacted PbI_2_. Moreover, the PL peak associated with the quasi-2D PEA_2_MA_*n*–1_Pb_*n*_I_3*n*+1_ phase with *n* = 2 increases with heating time for both MAPbI_3_ and MAFACsPbI_3_ samples, as shown in Figure S8 and Figure S9. To further study any variation within the bulk, the PL of PEAI-treated MAPbI_3_ and MAFACsPbI_3_ films was measured by exciting the material from the glass side before and after thermal aging for 6 h (Figure S10). As previously shown, PEAI-treated MAPbI_3_ shows a weak PL peak in the low-wavelength region already at time 0, which remains unaltered during thermal aging. In contrast, the PL peak at low wavelength appears in the PEAI-treated MAFACsPbI_3_ after 6 h of thermal aging at 85 °C, suggesting that the large cations have diffused through the material, forming quasi-2D structures close to the substrate’s interface. This is in agreement with the loss of PL intensity from the 2D feature close to the thin film surface.

In order to detect any structural change upon thermal exposure, we measured the XRD patterns before and after thermal aging (85° for 250 h in N_2_), which are shown in Figure 3c,d. Both materials initially showed a distinct peak at ∼4.7° assigned to the crystalline PEAI form, which totally disappeared after thermal aging. Upon thermal aging, a weak XRD peak at 4° assigned to the mixed 2D PEA_2_MA_*n*–1_Pb_*n*_I_3*n*+1_ phase with *n* = 2 appears, in agreement with PL results shown in Figure 3a. Most importantly, the intensity of XRD peaks typical of the 3D perovskite phase (14, 28, and 32°) decreases for both materials; i.e., the (110) peak at ∼14° is reduced by 42 and 26% for 2D/MAPbI_3_ and 2D/MAFACsPbI_3_, respectively. The results suggest that the 2D capping is not stable when exposed to thermal stress and undergoes structural transformations, which may lead to partial diffusion of the bulky molecule through the film. The degradation of the underneath 3D bulk is slowed down but not completely avoided when the material is subjected to long-term thermal aging; i.e., the 3D perovskite phase diffraction signal of 2D/MAFACsPbI_3_ is reduced by 26% with respect to the initial value, while it decreases by 35% in pristine MAFACsPbI_3_.

**Figure 4:**
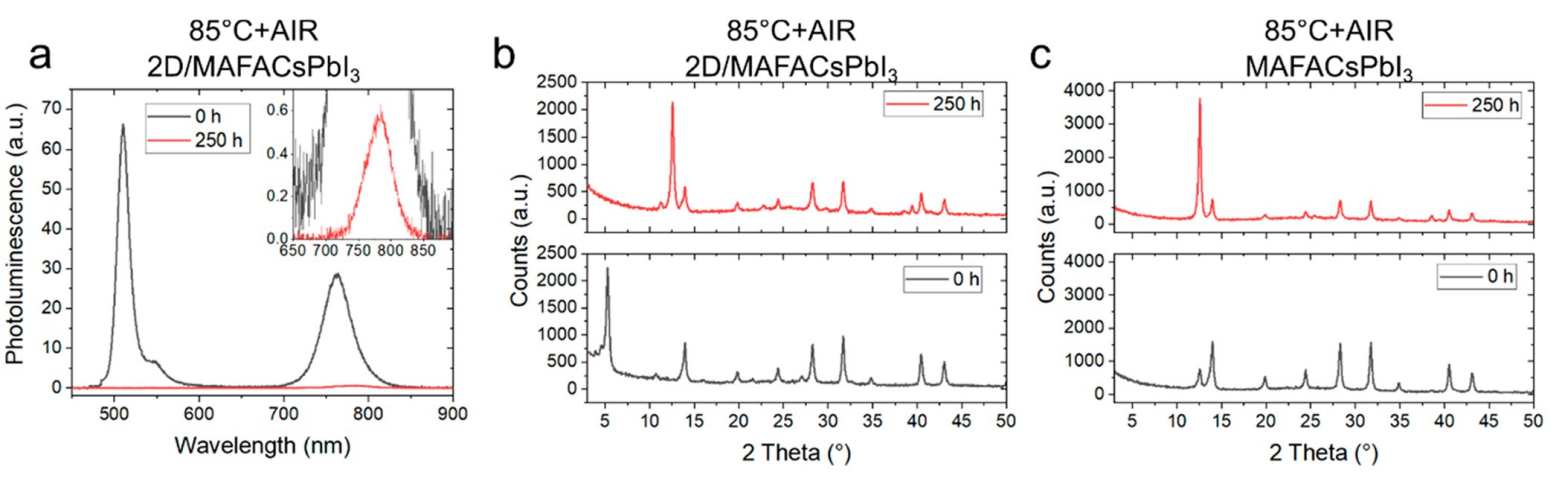
(a) PL spectra of PEAI-treated MAFACsPbI_3_ before and after thermal aging at 85 °C for 250 h in atmospheric air (RH 35%, CW 450 nm excitation). (b) XRD peaks of PEAI-treated MAFACsPbI_3_ before (bottom) and after (top) thermal aging at 85 °C for 250 h in atmospheric air. (c) XRD peaks of pristine MAFACsPbI_3_ before (bottom) and after (top) thermal aging at 85 °C for 250 h in atmospheric air.

To assess whether the PEAI-based 2D capping layer can effectively protect the underneath bulk from moisture, we have repeated the thermal aging experiments for PEAI-treated MAFACsPbI_3_ in air (85 °C, 250 h, air 35% relative humidity - RH). Figure 4a shows the PL spectra measured for the material excited from the film side before and after thermal exposure in air. The inset image displays a magnification of the PL peak in the 650–900 nm range. PL peaks associated with the pure PEA_2_PbI_4_ and quasi-2D phase completely disappear upon thermal aging, and the one assigned to the 3D bulk at 760 nm is considerably reduced. Similarly, the XRD peak at low angles typical of the 2D PEA_2_PbI_4_ phase disappears (Figure 4b), while the intensity of the XRD peaks assigned to the 3D perovskite structure decreases, leaving behind PbI_2_ as decomposition product, as evidenced by the intense XRD peak at 12.5°, which corresponds to the 001 peak of crystalline PbI_2_. We previously showed that the presence of a 2D capping layer seems to retard the thermal degradation of MAFACsPbI_3_ when aged in N_2_ at 85 °C. However, the presence of moisture accelerates the degradation of the 2D capping layer, with subsequent loss of the bulky cation, failing in protecting the 3D bulk from thermal stress and allowing moisture to penetrate through the material damaging it. Indeed, similar degradation of a pristine MAFACsPbI_3_ is observed when aged under the same conditions (Figure 4c). Figure S11 shows the XRD patterns of MAPbI_3_ and PEAI-treated MAPbI_3_ films as deposited and after being aged at 85 °C in air for 250 h (RH 35%). Similarly to what was observed for MAFACsPbI_3_, the presence of moisture accelerates the degradation of the 2D/3D heterojunction, allowing water molecules to penetrate through the film and convert MAPbI_3_ into PbI_2_. The presence of the bulky cation in the 2D capping layer can act as a barrier to prevent moisture uptake. However, prolonged exposure to moisture at high temperature (85 °C) may cause both a simple diffusion of the large cation and/or the volatilization of PEA and HI, causing the hydration of the 3D perovskite underneath. Our results show that PEA_2_PbI_4_ is not sufficient in protecting the underlying 3D perovskite from moisture uptake and degradation when it is coexposed to thermal stress.

To further evaluate the stability variations upon PEAI addition in MAPbI_3_ and MAFACsPbI_3_ thin film, we calculated the intensity proportion between the (001) peak of crystalline PbI_2_ and the (110) peak of the perovskite tetragonal phase before and after thermal aging in air for 250 h (Figure S12). The stability of MAFACsPbI_3_ is slightly enhanced when the surface is treated with PEAI, showing reduced conversion of halide perovskite into crystalline PbI_2_. In contrast, PEAI on MAPbI_3_ seems to negatively affect its stability, accelerating the degradation. This might be due to its fast penetration through the bulk and damage of the [PbI_6_]^4–^ structure, with subsequent acceleration of the 3D crystal structure disruption. Recently, Lei et al. observed that an excess of PEAI addition in MAPbI_3_ can indeed accelerate its degradation due to interaction between the amino group of PEA^+^ and the [PbI_6_]^4+^ octahedral, leading to the damage of the 3D crystal structure.^38^ Our results further support this observation, showing that the quality of the bulk of the 3D perovskite underneath the 2D capping layer will define also the degradation rate of the film.

**Figure 5:**
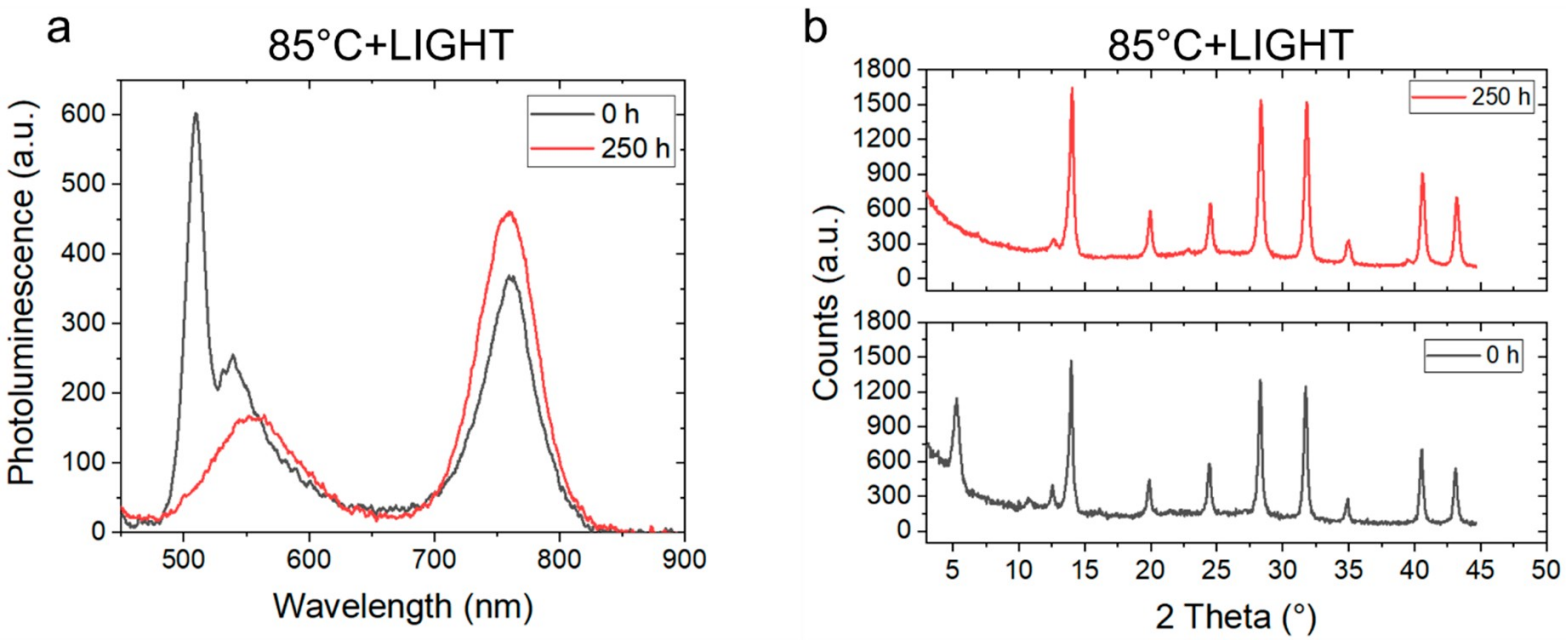
(a) PL spectra of PEAI-treated MAFACsPbI_3_ before and after thermal aging at 85 °C for 250 h in N_2_ under continuous simulated 1 sun illumination (CW 405 nm excitation). (b) XRD peaks of PEAI-treated MAFACsPbI_3_ before (bottom) and after (top) thermal aging at 85 °C for 250 h N_2_ under continuous simulated 1 sun illumination.

Finally, the thermal aging experiment for PEAI-treated MAFACsPbI_3_ was repeated under continuous illumination (85 °C, 250 h, N_2_ under simulated 1 sun illumination). Figure 5a shows the PL spectra of a PEAI-treated MAFACsPbI_3_ thin film before and after 250 h of thermal aging under continuous simulated 1 sun illumination in N_2_. While the peak centered at about 760 nm assigned to the 3D bulk remains unchanged, the pure 2D PEA_2_PbI_4_ phase disappears during the first 20 h of aging (see Figure S13), leaving behind a broad peak centered at about 560 nm, which suggests the evolution of the pure 2D phase into mixed PEA_2_MA_*n*–1_Pb_*n*_I_3*n*+1_ phases. Interestingly, such dynamical evolution of the 2D/3D multijunction was not observed when the film was thermally annealed in the dark (see Figure 3b). The XRD measurements shown in Figure 5b confirm the absence of a pure 2D PEA_2_PbI_4_ phase after 250 h of aging at 85 °C, in N_2_ under illumination and unaltered crystallinity of the underneath 3D bulk. Figure S14 shows the PL spectra of the PEAI-treated MAFACsPbI_3_ film measured from the glass side after thermal aging at 85 °C under continuous illumination (patterns plotted in logarithmic scale). We noticed the appearance of a peak at about 550 nm, indicating the formation of mixed PEA_2_MA_*n*–1_Pb_*n*_I_3*n*+1_ phases near the glass substrate. These results suggest that even MAFACsPbI_3_ films, structurally more stable and with a better optoelectronic quality, show the diffusion of PEAI through the 3D bulk under light soaking and thermal stress, which might have some implications on the stability of the material if further stressed. Similar dynamical evolution of the 2D/3D MAFACsPbI_3_ interface was observed when films were aged at room temperature (25 °C), in N_2_ under illumination (Figure S15 and Figure S16), showing that the dynamical structural variation of the 2D/3D multijunction occurs in the presence of light and is accelerated by temperature. Light-induced degradation of the PEA_2_PbI_4_ capping layer may be due to a slow and partial volatilization of PEA and HI within the 250 h of illumination, which are released from the surface, leaving PbI_2_ residue as previously reported by Fang et al.^39^ for PEA_2_PbI_4_ flakes. 2D perovskites suffer for the presence of defects and light instability—even more than 3D systems. Thus, here we learn that also in the form of a 2D/3D heterojunction they still retain most of the weaknesses identified for pure 2D thin films, flakes, or single crystals.

To conclude, we studied the 2D/3D multijunction formation by depositing PEAI in IPA on top of MAPbI_3_ and MAFACsPbI_3_. 2D PEA_2_PbI_4_ capping layers are formed on the surface of both 3D bulk perovskites. The capping layer firmly adheres on the surface of MAFACsPbI_3_. However, part of the PEAI might diffuse through the 3D bulk when subjected to thermal aging at 85 °C in N_2_ for 250 h, failing in protecting the underneath 3D bulk from degradation and causing loss in crystallinity. In contrast, PEAI partly diffuses through the bulk of MAPbI_3_ even before the film is subjected to thermal stress, probably due to its higher defectivity. We also show that the 2D capping layer degrades when the same thermal aging test is performed in air at 85 °C and the underneath 3D bulk irreversibly converts to PbI_2,_ irrespectively of the 3D bulk composition. Finally, we found that the 2D capping layer undergoes a structural evolution from pure to mixed 2D phase when the film is aged under simulated 1 sun illumination. Such structural evolution is accelerated when light soaking is combined with high temperature. Our results show that light soaking induces major dynamical structural changes of the 2D capping layer; this process is accelerated by temperature, strengthening the importance of evaluating the stability of PSCs under continuous illumination and at high temperatures in order to evaluate its proper implementation in a working device.

## Supporting Information Available

Additional figures, including UV–vis absorption spectra, PL spectra evolution during 250 h thermal aging, and XRD patterns before and after thermal aging and peak intensity study (PDF)
