# Supplementary material for: Thermal- and Light-Induced Evolution of the 2D/3D Interface in Lead-Halide Perovskite Films
Source: ACS Appl Mater Interfaces. 2021 Sep 29;14(30):34180–8. doi: 10.1021/acsami.1c09695 (PMC9354011; doi:10.1021/acsami.1c09695)
Supplement: Supplementary file 1 — am1c09695_si_001.pdf [file am1c09695_si_001.pdf]

Supporting Information

Thermal- and Light-Induced Evolution of the 2D/3D Interface in Lead-Halide Perovskite Films

Francesca Fiorentino[1,2], Munirah D. Albaqami[3], Isabella Poli[1], Annamaria Petrozza[1,3*]*

1. Center for Nano Science and Technology @PoliMi, Istituto Italiano di Tecnologia, via G. Pascoli 70/3, 20133, Milano, Italy.

2. Physics Department, Politecnico di Milano, Piazza L. da Vinci, 32, 20133 Milano, Italy.

3. Chemistry Department, College of Science, King Saud University, Riyadh 11451, Saudi Arabia

e-mail: annamaria.petrozza@iit.it

e-mail: isabella.poli@iit.it

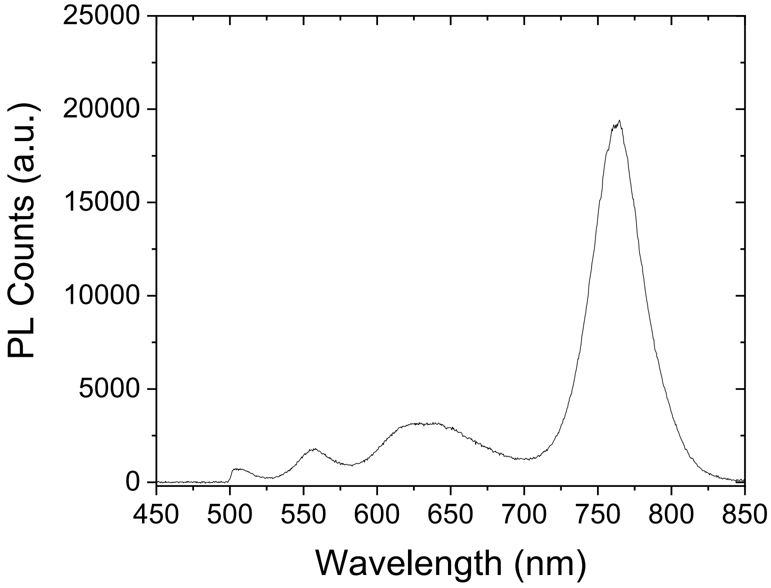

Figure S1. PL spectrum of PEAI-treated $MAFACsPbI_3$ without PbI_2 excess.

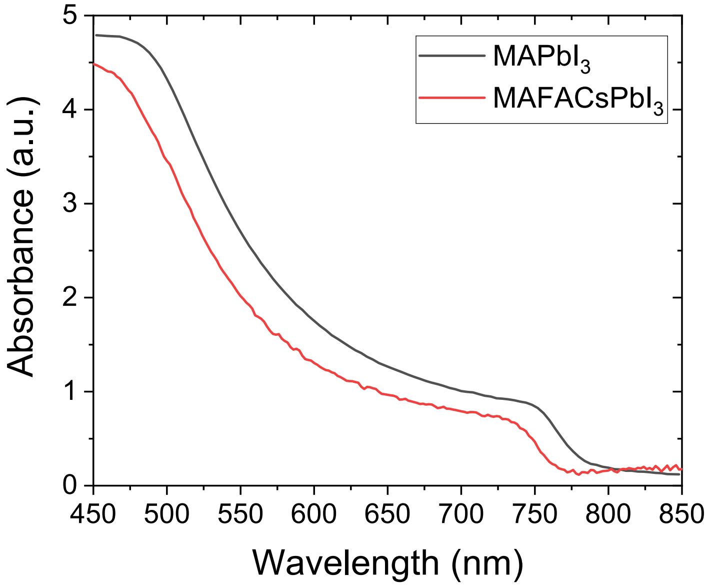

Figure S2. UV-Vis absorbance of $MAPbI_3$ and $MAFACsPbI_3$ thin films.

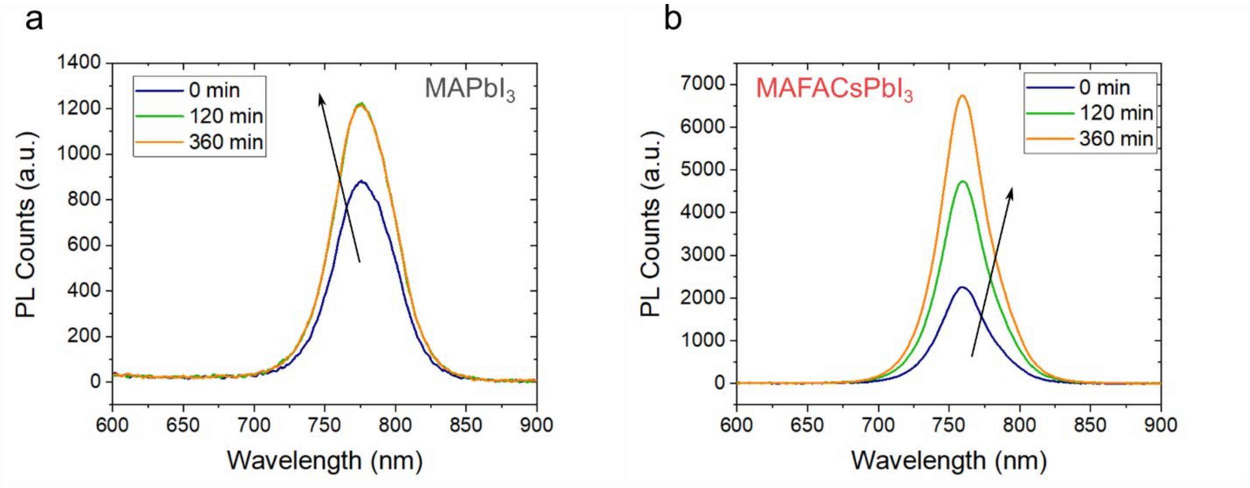

Figure S3. a-b PL spectra of $MAPbI_3$ and $MAFACsPbI_3$ thin films measured in air (RH 35%) at room temperature over 6 hours, respectively.

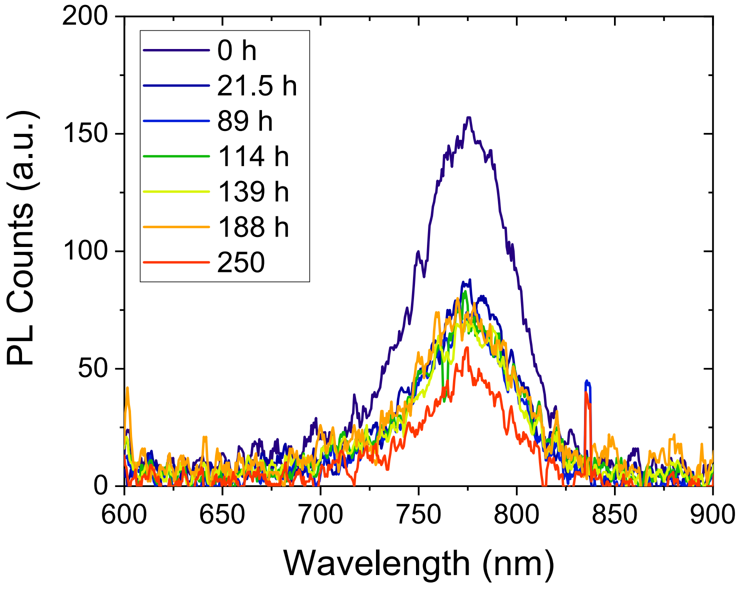

Figure S4. PL spectra of $MAPbI_3$ under thermal stress at 85°C in N_2 for 250 h.

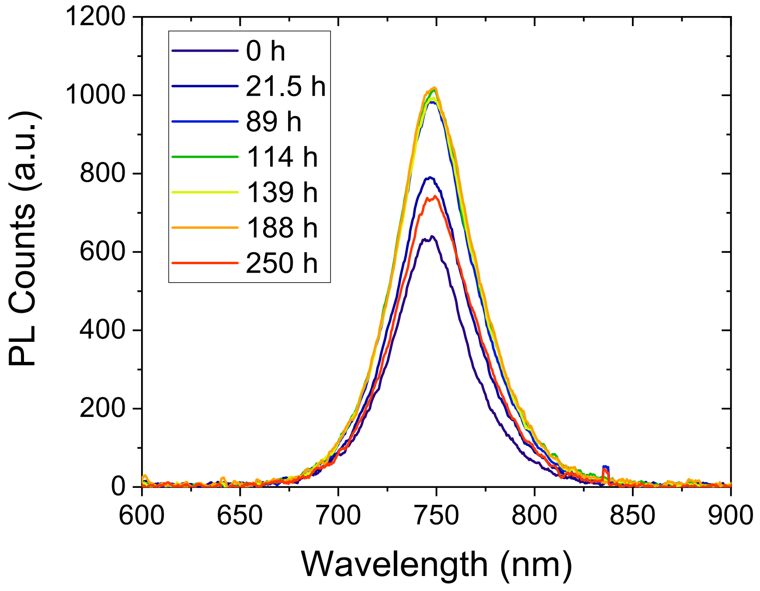

Figure S5. PL spectra of $MAFACsPbI_3$ under thermal stress at 85°C in N_2 for 250 h.

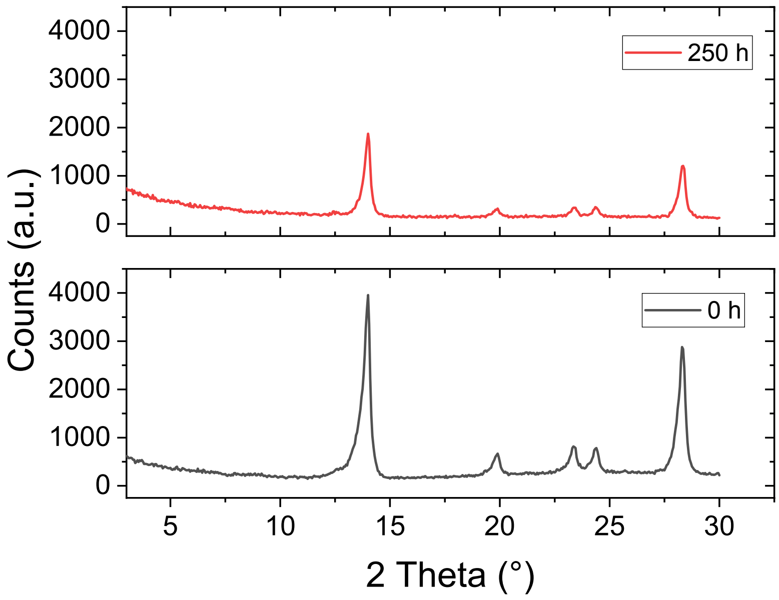

Figure S6. XRD patterns of $MAPbI_3$ before and after thermal ageing at 85°C in N_2 for 250 h. The intensity of XRD peaks gets halved under thermal stress, indicating reduced crystallinity.

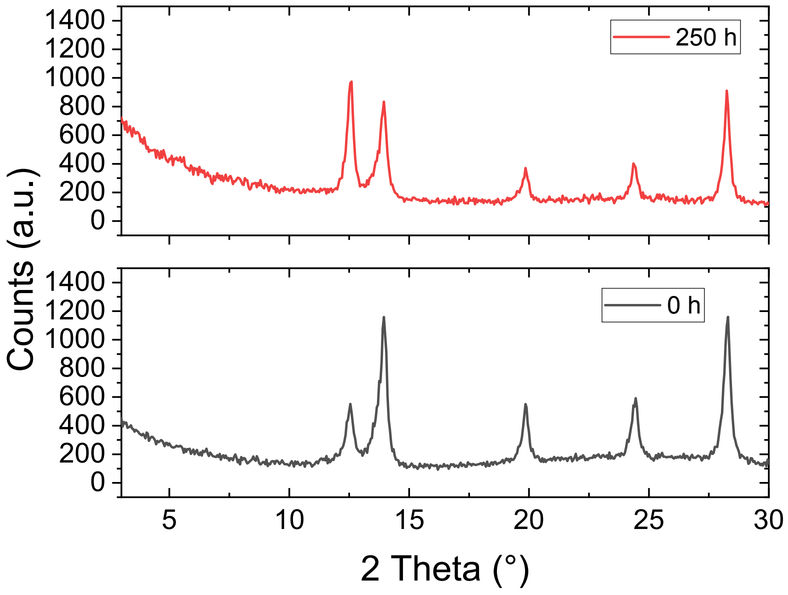

Figure S7. XRD patterns of $MAFACsPbI_3$ before and after thermal ageing at 85°C in N_2 for 250 h. The intensity of XRD peaks associated to the 3D perovskite phase reduce by 35% with respect the initial values under thermal stress, indicating lower crystallinity.

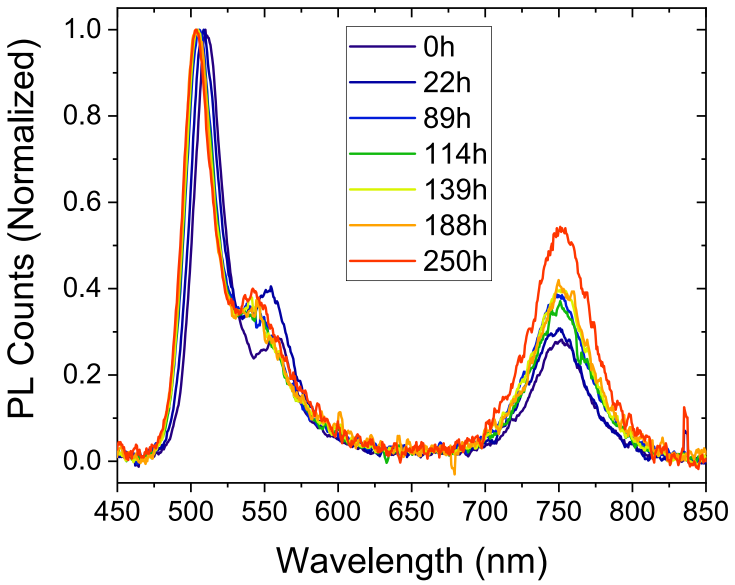

Figure S8. Dynamic evolution of PL spectrum of PEAI-treated $MAFACsPbI_3$ upon thermal ageing at 85 °C in N_2 for 250 hours. Spectra have been normalized with respect to the 2D peak n=1 at 520 nm.

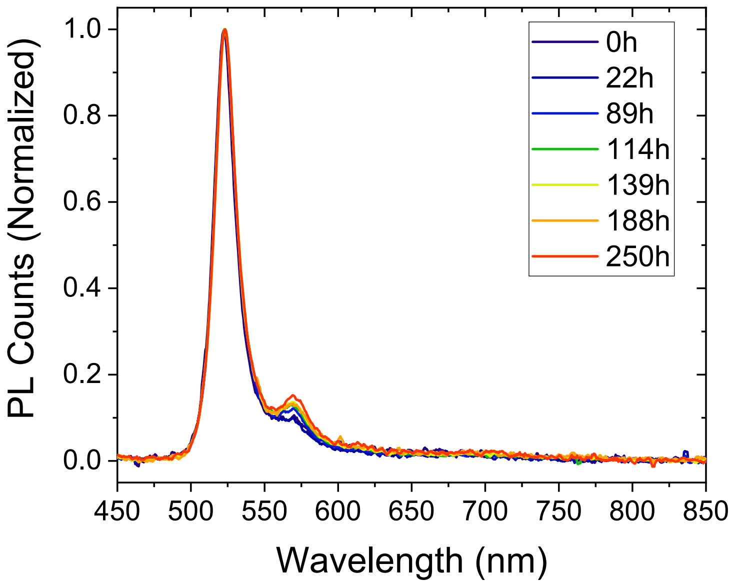

Figure S9. Dynamic evolution of PL spectrum of PEAI-treated $MAPbI_3$ upon thermal ageing at 85 °C in N_2 for 250 hours. Spectra have been normalized with respect to the 2D peak n=1 at 525 nm.

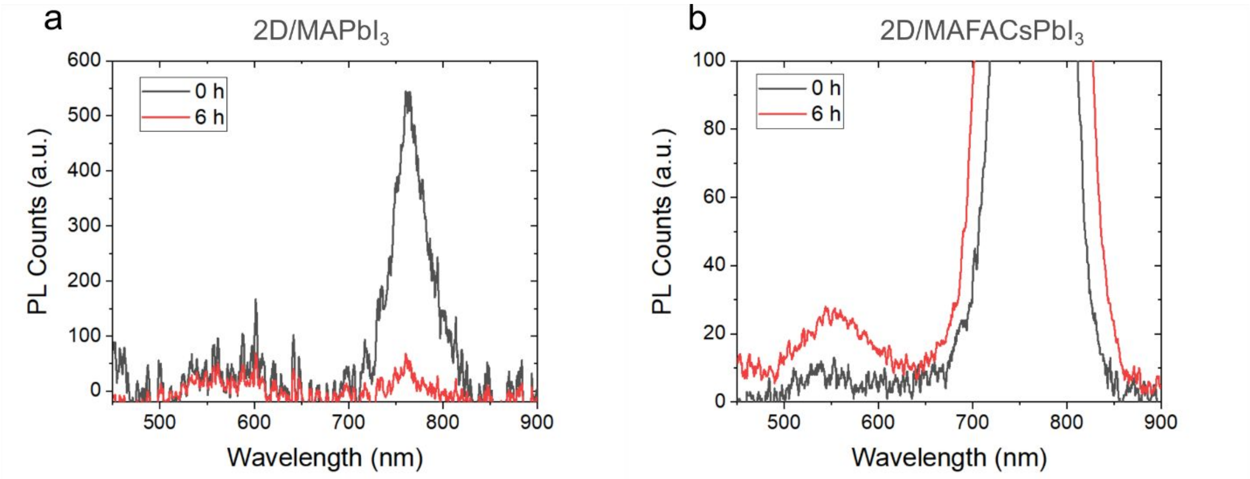

Figure S10. a-b PL spectra of 2D/$MAPbI_3$ and 2D/MAFACsPbI3 films measured by exciting the film from the glass side (CW 405 nm, PL acquired at room temperature) before and after thermal ageing at 85°C for 6 hours.

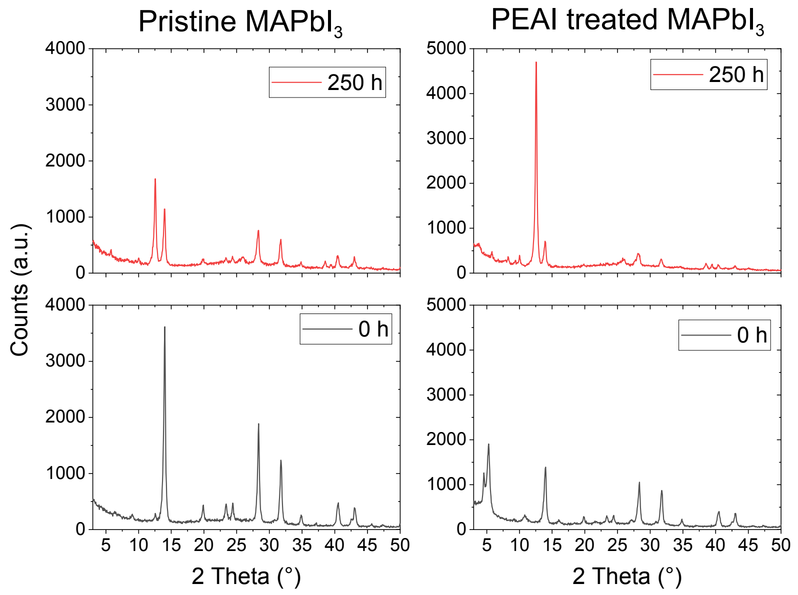

Figure S11. XRD patterns of pristine $MAPbI_3$ (left panels) and PEAI-treated $MAPbI_3$ (right panels) before and after 250 hours of thermal ageing at 85°C in air with RH 35% (black and red lines, respectively).

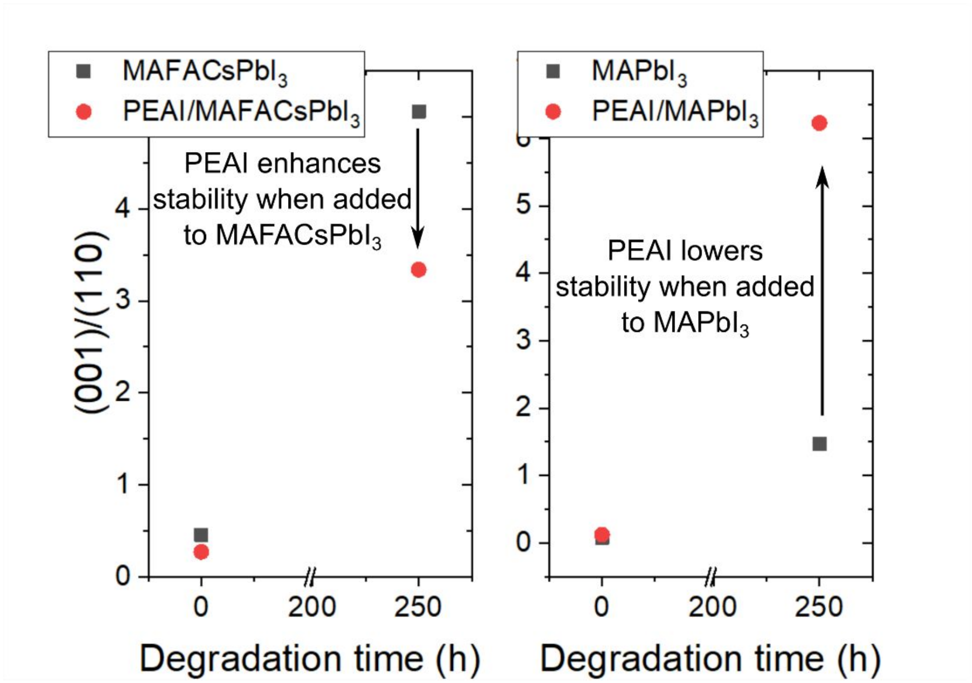

Figure S12. Intensity proportion between the (001) peak of PbI_2 and (110) peak of perovskite before and after thermal ageing treatment at 85°C in air (RH 35%).

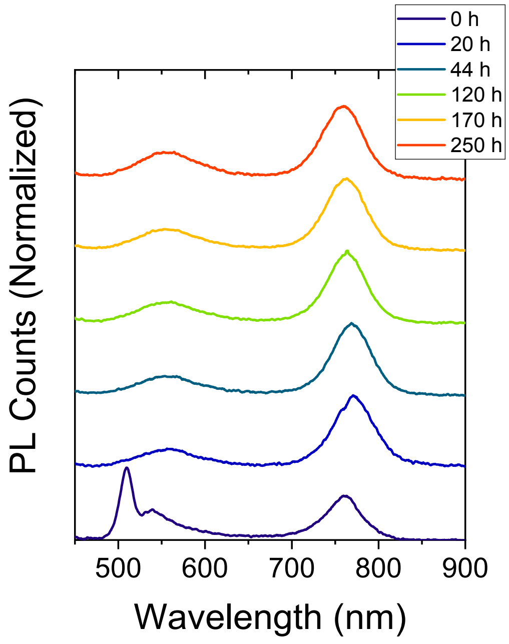

Figure S13. Evolution of PL spectra of PEAI-capped $MAFACsPbI_3$ thin films under thermal ageing at 85°C in N_2 under continuous illumination for 250 hours.

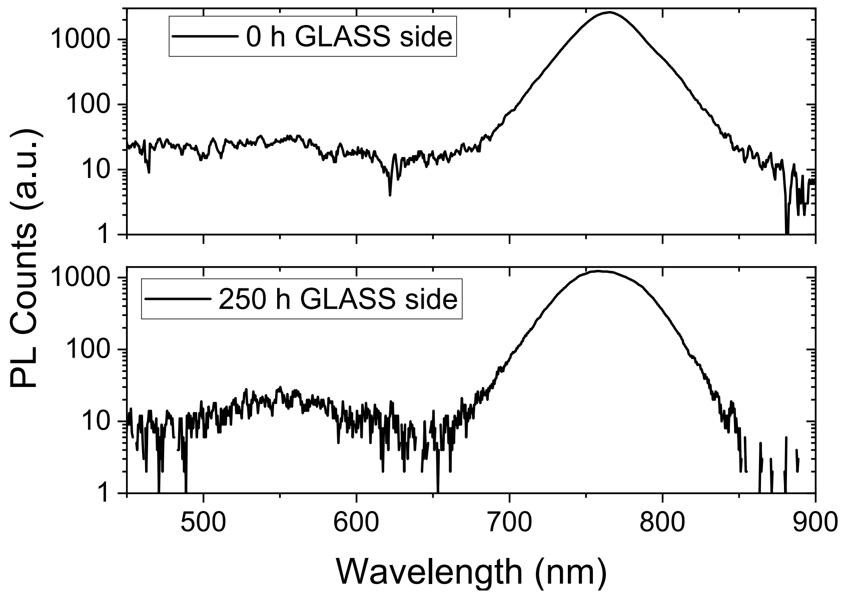

Figure S14. PL spectrum evolution of PEAI-capped $MAFACsPbI_3$ thin films before and after thermal ageing at 85°C for 250 hours under continuous simulated 1 sun illumination in N_2. PL spectrum measured using a 405 nm laser excitation by the glass side.

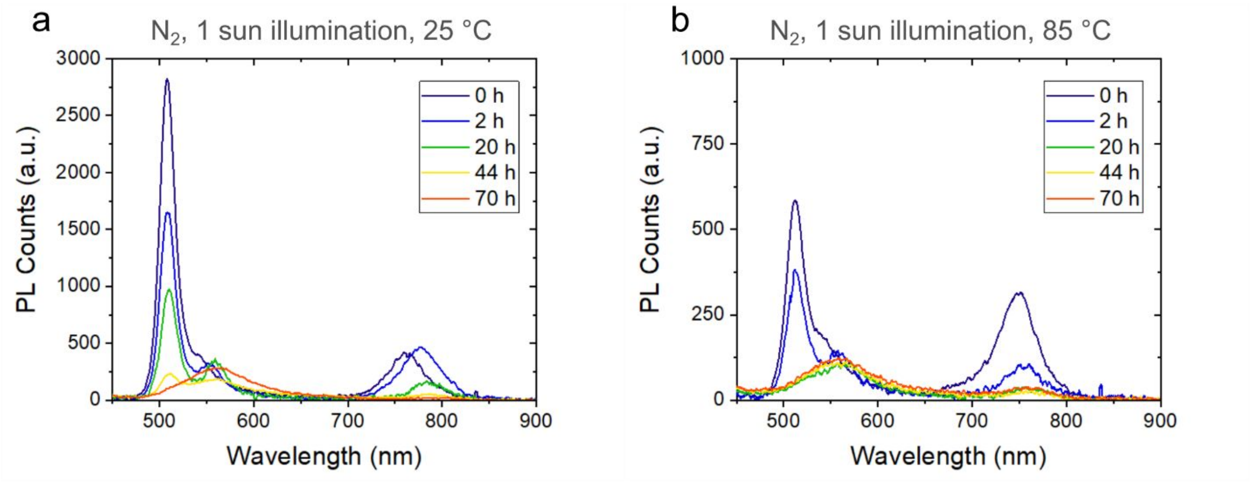

Figure S15. a-b PL spectra of PEAI-treated MAFACsPbI3 films aged under continuous illumination (simulated 1 sun), in N2 and at 25°C and 85°C, respectively (CW 405 nm illumination).

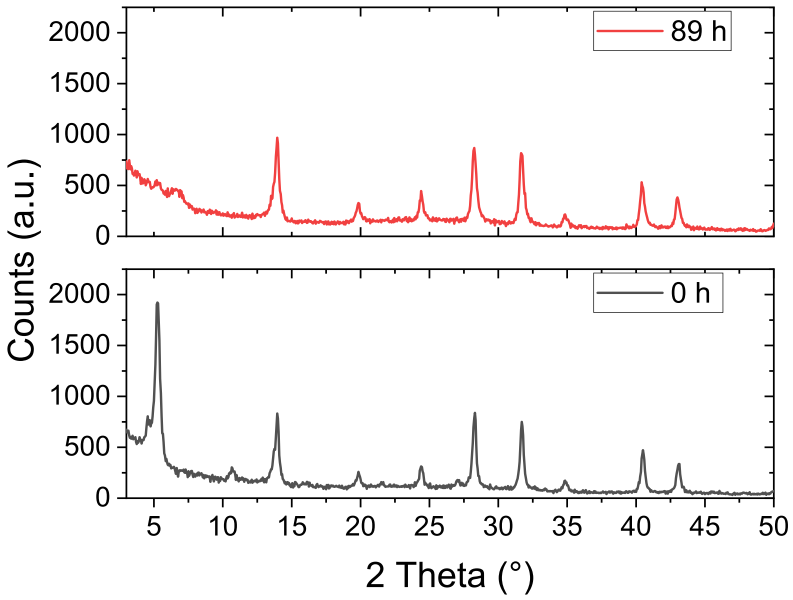

Figure S16. XRD patterns of $MAFACsPbI_3$ before (bottom) and after (top) light soaking in N_2 at room temperature (simulated 1 sun illumination) for 89 hours.
